# Differences among fathers, mothers, and teachers in symptom assessment of ADHD patients

**DOI:** 10.3389/fpsyt.2023.1029672

**Published:** 2023-06-23

**Authors:** Xia Huang, Hui-Qin Li, Alan Simpson, Jia-Jun Xu, Wan-Jie Tang, Yuan-Yuan Li

**Affiliations:** ^1^Mental Health Center, West China Hospital, Sichuan University/West China School of Nursing, Sichuan University, Chengdu, Sichuan, China; ^2^Mental Health Center, West China Hospital, Sichuan University, Chengdu, Sichuan, China; ^3^Florence Nightingale Faculty of Nursing, Midwifery and Palliative Care and Health Services and Population Research Institute of Psychiatry, Psychology and Neuroscience King’s College London, London, United Kingdom

**Keywords:** ADHD, SNAP-IV, attention deficit hyperactivity disorder, symptom assessment, child psychiatry (ADHD)

## Abstract

**Background:**

The Swanson Nolan, and Pelham scale version IV (SNAP-IV) is the most critical tool for ADHD screening and diagnosis, which has two scoring methods. ADHD requires symptom assessment in multiple scenarios, and parent and teacher reports are indispensable for diagnosing ADHD. But the differences of assessment results from fathers, mothers and teachers, and the consistency of results from different scoring methods are unknown. Therefore, we carried out this study to understand the differences in the scores of fathers, mothers and teachers using SNAP-IV for children with ADHD and to explore the differences in scoring results under different scoring methods.

**Methods:**

The SNAP-IV scale and Demographics Questionnaire and Familiarity Index were used to survey fathers, mothers and head teachers. Measurement data are expressed as the mean ± standard deviation (x ± s). The enumeration data were described by frequency and percentage. ANOVA was used to compare group differences in mothers’, fathers’, and teachers’ mean SNAP-IV scores. The Bonferroni method was used for *post hoc* multiple comparison tests. Cochran’s Q test was used to compare the differences in the abnormal rate of SNAP-IV score results of mothers, fathers and teachers. Dunn’s test was used for *post hoc* multiple comparison tests.

**Results:**

There were differences in scores among the three groups, and the differences showed inconsistent trends across the different subscales. Differences between groups were calculated again with familiarity as a control variable. The results showed the familiarity of parents and teachers with the patients did not affect the differences in their scores. The evaluation results were different under two assessment methods.

**Conclusion:**

Results concluded that fathers did not appear to be an appropriate candidate for evaluation. When using the SNAP-V for assessment, it should be comprehensively considered from both the scorer and symptom dimensions.

## Highlights

- Both parent and teacher reports are indispensable for diagnosing ADHD. users of DSM-5 need to know if and in what way differences exist between the different raters where each rater is located.
- In terms of the average and total scores of fathers, mothers, and teachers on the subscales, there were differences in scores among the three groups, and the differences showed inconsistent trends across the different subscales.
- The familiarity of parents and teachers with the patients did not affect the differences in their scores.
- Teachers were more positive than fathers in both ANI and H/I and higher than mothers in H/I.
- Different scoring methods may result in different assessments of abnormality.

## Introduction

Attention deficit hyperactivity disorder (ADHD) is a common psychobehavioral problem in childhood. The global prevalence of ADHD in children is 5% (1), and the prevalence of ADHD in Chinese children and adolescents is 6.3% (2). ADHD will have persistent and extensive negative effects on individuals’ cognition, behavior, and social function, bringing a heavy burden to the family and society (3–5). A study stated ADHD to be associated with poorer performance on working memory, tasks measuring inhibition, vigilance, and planning (6). Undiagnosed and untreated ADHD has serious consequences for patients. It not only has a dramatic negative impact on children’s academic performance (7, 8), but may also lead to an increased risk of other mental health problems, such as personality disorders (9), psychiatric disorders (10), and substance abuse (5). In addition, a systematic review showed that children diagnosed with ADHD had a higher risk of suicidal behavior later (11). It is a severe public health problem that needs attention (12).

Early screening and diagnosis of ADHD are essential for treatment and rehabilitation. The diagnosis of ADHD can be complicated by overlapping symptoms between ADHD and other underlying comorbid psychiatric disorders (13, 14). Rating scales are valuable tools for measuring ADHD symptoms. The Swanson Nolan, and Pelham scale version IV (SNAP-IV) is a DSM symptom-based scoring scale that is the most critical tool for ADHD screening and diagnosis, has been translated by many countries and is widely used worldwide (15–17). The scale includes 18 items assessing symptoms of ADHD and 8 items assessing symptoms of oppositional defiant disorder (ODD), which constituted the three factors of the scale (inattention-INA, hyperactivity/impulsivity-H/I, oppositional defiant disorder-OD). The scale’s structural validity was demonstrated in community children and clinical samples from the United States (18), Taiwan (15), Japan (16), and Brazil (19). SNAP-IV as a screening tool showed 82.3% sensitivity and 82.4% specificity in assessing ADHD clinical symptoms with physicians (20). This scale’s good reliability and validity make it a common and vital tool for ADHD screening and diagnosis, and it is widely used in clinical practice.

When doctors use SNAP-IV to diagnose children, parents and teachers are the usual reporters of ADHD symptoms. However, for a variety of reasons, face-to-face conversations usually involve only one guardian, So many doctors only gets information from one parent and ignores the teacher and the other parent. The fifth edition of the Diagnostic and Statistical Manual of Mental Disorders (DSM-5) proposes that ADHD requires symptom assessment in multiple scenarios. Both parent and teacher reports are indispensable for diagnosing ADHD (21). A single source of information and scenarios in the clinic are inconsistent with *DSM-5* requirements. However, it is understandable that obtaining sufficient information from teachers and both parents, may present logistical challenges for busy medical staff. After all, bringing a teacher to the diagnostic room is not an easy task, and when both parents as a whole make a unique assessment of symptoms, information from one parent may be overlooked. Therefore, to interpret scores more accurately, users of the scale need to know if and in what way differences exist between the different raters where each rater is located.

Many previous studies have explored the effectiveness of parents (19, 22) or teachers (23) in assessing ADHD using the SNAP-IV scale. Only a few studies have examined differences in parental and teacher assessments (18, 19, 24, 25), and no studies have compared differences between different parents. Among them, Anne Karin’s study (24) confirmed the difference between parents and teachers in assessing ADHD prevalence in children. Swanson’s study (18) believed that only parental scores could effectively distinguish high-risk children who meet the DSM-IV Stage II ADHD criteria. Maria Antonia Serra-Pinheiro (25) explored differences in parental and teacher assessments of boys and girls. On the subscale, teachers and parents had different scoring characteristics in their assessment of boys and girls. Unfortunately, these studies have the following problems: 1. The sample is community children, and there are no evaluation data of parents and teachers of children with clinical ADHD (18, 25), 2. Only one assessment method was used for assessment (18, 25), 3. The studies were conducted in developed countries, and there are no data from developing countries (18, 19, 24, 25), 4. Parents often evaluate ADHD as a whole. No studies have compared the assessment results of fathers, mothers and teachers separately (18, 19, 24, 25).

In addition, SMAP-IV has two scoring methods. One way is to calculate the average item score representing the severity of symptoms. The researchers tested cut points in different populations as clinical diagnostic recommendations. Another way is to count the number of items greater than 1 in each subscale and make recommendations for “abnormal” according to the ADHD symptom item criteria in the DSM-V. A study (26) explored the differences between different scoring methods in the relief of symptoms of ADHD. However, no study has explored whether different scoring methods are consistent in the screening and diagnosing of ADHD. Based on the above, we make the following assumptions: 1. The symptoms of ADHD children in clinical samples are more easily recognized; There are differences in the assessment of fathers, mothers, and teachers; 3. There are differences in the evaluation results under different evaluation methods. To verify the above assumptions, we carried out this study to understand the differences in the scores of fathers, mothers and teachers using SNAP-IV for children with ADHD and to explore the differences in scoring results under different scoring methods to provide a reference for the early screening and diagnosis of ADHD.

## Method

### Subjects

The subjects of this study were fathers, mothers and headteachers of ADHD patients diagnosed using DSM-5. The patients ranged in age from 6 to 17 years and were diagnosed with ADHD by psychiatrists with doctoral degrees and more than a decade of work experience. The following patients were excluded: (1) Combined with other mental disorders, (2) Divorce or separation of parents, (3) Death of one (both) parent (s), (4) Patients were not attending school (including kindergarten), and (5) Combined diseases of the nervous system, such as autism spectrum disorder, developmental learning disorder and developmental coordination disorder.

### Data collection procedures

We recruited samples from the outpatient department of the Mental Health Center, West China Hospital, Sichuan University. A convenience sampling method was used to enroll ADHD patients who met the inclusion and exclusion criteria. Parents of patients with ADHD are invited to complete the electronic SNAP-IV. The study obtained the patient’s headteacher’s WeChat (similar to Facebook) through parents. An email was sent to the patient’s head teacher. The email contained the introduction of the purpose and content of the study, the electronic version of the questionnaire, and the requirements for filling in the questionnaire, and the head teacher was invited to fill in the questionnaire. The time interval between the parents and the headteacher completing the questionnaire was within 3 days, and both used two assessment methods to evaluate the patients. The data collection period was from October 2019 to October 2021.

### Measures

#### SNAP-IV

The SNAP-IV scale uses Lanting Guo’s version, Chinese-translated from the MTA version (27). The internal consistency reliability of the parental version in China was Cronbach’s alpha of 0.95. The Cronbach’s alpha coefficients of the three subscales of INA, H/I and ODD were 0.90, 0.89 and 0.88, respectively. The intraclass correlation coefficient (ICC) of the test–retest reliability was 0.68. The test–retest reliability ICCs of the three subscales were 0.75, 0.76, and 0.24. The sensitivity was 0.87, and the specificity was 0.79 (28). The 26 items of the MTA SNAP-IV include the 18 ADHD symptoms specified in the DSM-IV (9 for INA and 9 for H/I) and 8 ODD symptoms. Items are rated on a 4-point scale ranging from (0) “not at all” to (3) “very much.”

Average Per Item Rating (ARI) subscale scores for the parent and teacher scales were calculated separately for the three subscales, resulting in nine SNAP-IV subscale scores ranging from 0 to 3. The scale scores were obtained from fathers, mothers and headteachers, abbreviated as F-Inatt, F-Hyp/Imp, F-Odd, M-Inatt, M-Hyp/Imp, M-Odd, T-Inatt, T-Hyp/Imp and T-Odd, respectively.

In this study, SNAP-IV scale was used to evaluate patients based on two different assessment methods. One is to calculate the average score across relevant items. A higher average score means more severe symptoms. Another way is to count the number of items greater than or equal to 2 in each subscale to judge whether it is “abnormal.” For the Inatt and Hyp/Imp subscales, the result is defined as “abnormal” if the number of items with a score greater than 1 is more than 6. For the Odd subscale, the result is defined as “abnormal” if the number of items with a score greater than 1 is more than 4. In the overall evaluation, if any of the subscales was abnormal, it was defined as the overall abnormality.

#### Demographics questionnaire and familiarity index

To explore the influence of personal characteristics on the results, we collected participants’ gender and age. In addition, parents and teachers were asked to rate their familiarity with the patients on a scale of 1 = Extremely familiar, 2 = Somewhat familiar, and 3 = Not at all familiar.

### Data analysis

Measurement data are expressed as the mean ± standard deviation (x ± s). The enumeration data were described by frequency and percentage, and the χ2 test was used to compare the gender distribution differences in different age groups. ANOVA was used to compare group differences in mothers’, fathers’, and teachers’ mean SNAP-IV scores. The Bonferroni method was used for *post hoc* multiple comparison tests. Analysis of covariance was used to compare group differences between mothers, fathers, and teachers, using familiarity (1 = Somewhat familiar, 2 = Extremely familiar) as covariances to control for their effects on ratings. Cochran’s Q test was used to compare the differences in the abnormal rate of SNAP-IV score results of mothers, fathers and teachers. Dunn’s test was used for *post hoc* multiple comparison tests. The Fleiss Kappa coefficients of the three groups were calculated.

### Ethical considerations

The Ethics Committee of Sichuan University approved this study. Before filling out the questionnaire, the investigator introduced the questionnaire’s purpose, significance, and requirements. Electronic versions of notification information and informed consent, as well as questionnaires, were sent through WeChat. Written informed consent was obtained from the respondents. Respondents understand that they have the right to withdraw at any stage of the questionnaire. The names of all respondents were not recorded, and all information about respondents will be kept confidential and used for scientific research only. In addition, the head teachers are required to keep all the information of the patients confidential so as not to bring trouble to the patients. No identifying information is presented in this paper.

## Results

### Demographic characteristics

Fathers, mothers and teachers of 336 ADHD patients completed the questionnaire. The age distribution of patients was 6–17 years old (M = 8.88, SD = 2.073), of which 86.61% (291/336) were 6–11 years old, and 13.39 (45/336) were 12–17 years old. Males accounted for 82.44% (277/336). There was no difference in the gender distribution of different age groups, χ2 = 0.125, *p* = 0.723. Six head-teachers and three fathers stated they were completely unaware of the patient’s situation. Without knowing the patient at all, it is impossible to accurately evaluate the patient. Therefore, all assessment data for these nine patients are excluded (including assessments by teachers, fathers, and mothers). Finally, assessment data from 327 patients were included. The effective response rate is 97.32% in our study. Among the differences in familiarity, 83.06% (309/327) of mothers said they were very familiar, 65.32% (243/327) of fathers said they were very familiar, and only 19.89% (74/327) of teachers said they were very familiar with patients. The Friedman test was used to compare the familiarity of the three groups of evaluators, and the results showed that the familiarity was different (*p* = 0.000).

### SNAP-IV ratings of patients by parents and teachers (average score)

Table 1 reports the average and total scores of fathers, mothers, and teachers on the subscales. Overall, there were differences in scores among the three groups, and the differences showed inconsistent across the different subscales (symptom groups). For ANI scores, teachers ranked first (M = 1.73, SD = 0.75), followed by mothers (M = 1.69, SD = 0.63) and fathers (M = 1.55, SD = 0.61), and *post hoc* tests showed significant differences. In terms of H/I, teachers (M = 1.16, SD = 0.78) had significantly higher mean scores than fathers (M = 1.04, SD = 0.59). The mother’s evaluation was not significantly different from the other two groups. In terms of ODD, parents scored significantly higher than teachers (M = 0.90, SD = 0.74), while mothers (M = 1.16, SD = 0.61) scored higher than fathers (M = 1.02, SD = 0.55). Regarding the total score, the score of mothers (M = 1.32, SD = 0.50) was significantly higher than that of fathers (M = 1.21, SD = 0.49), while the difference between parents and teachers was not significant. It is worth mentioning that the ICC values of both the total score and the subscales were low, with H/I having the highest ICC value (0.360) and ANI the lowest (0.341).

**Table 1:** SNAP-IV ratings of patients by parents and teachers (average score).

	Fathers	Mothers	Teachers	F (sig)	*Post Hoc* tests	ICC	95%CL
ANI	1.55 (0.61)	1.69 (0.63)	1.73 (0.75)	9.642 (0.000)	teachers > mothers > fathers	0.341	0.272–0.411
H/I	1.04 (0.59)	1.09 (0.60)	1.16 (0.78)	4.159 (0.016)	teachers > fathers	0.390	0.322–0.458
ODD	1.02 (0.55)	1.16 (0.61)	0.90 (0.74)	20.052 (0.000)	mothers > fathers > teachers	0.360	0.289–0.432
TOTAL	1.21 (0.49)	1.32 (0.50)	1.28 (0.63)	4.789 (0.009)	mothers > fathers	0.350	0.281–0.419

### The effect of familiarity on ratings

Differences between groups were calculated again with familiarity (only including very familiar and general familiarity) as a control variable, and the results are shown in Table 2. The results showed that in each subscale and overall score, there was no statistically significant difference in scores between different familiarity groups, with P greater than 0.05, indicating that familiarity did not affect the scoring results. However, there was a statistically significant difference between different relationship scoring groups (*p* < 0.05), and differences in scores between different relationships still existed. Explain that the differences in scores for different relationships are not due to differences in source and familiarity.

**Table 2:** Covariance analysis results.

	Relationship F (sig)	Patient F (sig)	Familiarity F (sig)
ANI	9.808 (0.000)	2.596 (0.000)	0.887 (0.347)
H/I	3.240 (0.040)	2.933 (0.000)	0.062 (0.803)
ODD	7.844 (0.000)	2.792 (0.000)	1.099 (0.295)
TOTAL	4.745 (0.009)	2.632 (0.000)	0.768 (0.381)

### SNAP-IV scoring of patients by parents and teachers (item scoring)

The results using item scoring are shown in Table 3. In ANI, the abnormal score rate was 48.5% (159/327) for teachers and 38.5% (126/327) for fathers. *Post hoc* tests showed that teachers had a significantly higher abnormal rate than fathers. In H/I, the abnormal rate of teacher scores was significantly higher in 24.4% (80/327) than in fathers with 14.0% (46/327) and mothers with 15.2% (50/327). In terms of ODD, the abnormal rate of mothers was 30.5% (100/327), while the abnormal rates of fathers and teachers were only 24.4% (80/327) and 24.1% (79/327), respectively, but the difference between the three groups was not significant. For the total score, the abnormal rate was 89.3% (293/327) for mothers, 57.6% (189/327) and 48.5% (159/327) for teachers and fathers. The difference between the three groups was significant.

**Table 3:** SNAP-IV scoring of patients by parents and teachers (item scoring).

	Fathers	Mothers	Teachers	F (sig)	*Post Hoc* tests	Fleiss kappa	95%Cl
ANI	38.5% (126/327)	45.1% (148/327)	48.5% (159/327)	9.011 (0.011)	teachers > fathers	0.223	0.221–0.225
H/I	14.0% (46/327)	15.2% (50/327)	24.4% (80/327)	17.559 (0.000)	teachers > fathers teachers > mothers	0.183	0.181–0.185
ODD	24.4% (80/327)	30.5% (100/327)	24.1% (79/327)	5.613 (0.060)		0.213	0.211–0.215
TOTAL	48.5% (159)	89.3% (293)	57.6 (189)	29.810 (0.000)	mothers > teachers > fathers	0.109	0.107–0.111

## Discussion

The study found that the scores of fathers, mothers and teachers were different. Pappas (29) reported that the behavioral characteristics of ADHD are different at school and home. Given that school is a more structured environment, attention problems may be seen as noncompliance (30). Issues with academic performance deficits, organizational skills, and disruptive behavior are critical in schools (31) but may be less relevant in the home environment. The parents and teachers are located at home and school, respectively, which may be the reason for the difference in the scores of parents and teachers.

Bussing (18) showed that parental ANI scores over 1.8 and H/I scores over 2.4 could predict ADHD diagnosis, but there was no relationship between teacher scores and ADHD diagnosis. Unlike Bussing’s findings, our study showed that teachers were more positive than fathers in both ANI and H/I and higher than mothers in H/I. This discrepancy may be because Bussing’s study included community samples, whereas ours was a clinical sample. Symptoms of ANI and H/I in clinical patients may be more evident in school, so teachers have a better opportunity to detect and assess these two aspects than parents. Other studies also support that teachers’ information is valuable (32–34). These studies show that teachers have daily contact with children and have a wealth of experience with developmentally appropriate behaviors. Teacher assessments can serve as good ANI and H/I screening and diagnostic references.

Regarding the differences between parents, a possible reason is the difference in time and content of Chinese parents’ participation in family education (35). According to a survey, mainland Chinese fathers spend 0.92 h on childcare, while mothers spend 3.05 h (36). Gender differences in parental engagement also persisted: Chinese fathers preferred activities that were elicited to be pleasurable, supportive and interactive while leaving repetitive, time-consuming, and time-inflexible tasks to mothers (37). This difference in childrearing time and content may result in differences in children’s behavior and parents’ feelings. Overall, the maternal assessment had the highest positivity rate and appeared to be closest to the clinician’s judgment. If used for screening in a community population, the mother appears to be an optimal evaluator. However, the validation of this scenario was beyond the scope of our study, and this may be a direction for future research to explore.

The differences for each subscale are different. For ODD, fathers and mothers scored higher than teachers, possibly because children’s behavioral performance in oppositional defiance is more likely to be presented to parents. In China, because of the influence of Confucian culture, teachers are greatly respected in society. When reflected in the teacher-student relationship, it is manifested as the great authority of teachers in front of students (38, 39)^.^ Children may be less antagonistic to teachers than parents. In addition, as a place for collective activities, uniform discipline and behavioral norms are essential in schools where impulsive and destructive behaviors are considered unacceptable (31). Because these behaviors increase the difficulty of classroom management for teachers, their feelings are more pronounced.

Our findings suggest that, other than complete unfamiliarity, parental and teacher familiarity with the patient does not affect their assessment of the patient’s symptoms. This means that the clinical symptoms of ADHD can be recognized by people who are somewhat familiar with the patient in life and school. This suggests that it seems feasible to develop other populations such as classmates, sisters, brothers, etc. of the patient as evaluators. The assessment differences were more likely to arise from familiarity than the child’s relationship with the assessor. Additionally, it is the performance of the patient’s behavior in different scenarios rather than the recognition. To a certain extent, this allows more people to assess the ADHD symptoms of patients, such as the patient’s siblings and classmates. Future scholars can develop more ADHD scale versions suitable for other scenarios.

For the three groups of raters, the results scored by item were similar to the average score results. However, compared with the average scoring method, there was no difference between mothers and fathers in ANI, and there was a difference between teachers and mothers in H/I. In contrast, the difference in ODD did not show statistical significance. It is suggested that different scoring methods may result in different assessments of abnormality. Therefore, in practical applications, combining the two scoring methods may provide more reference for the clinical diagnosis of ADHD.

Since the evaluators are the parents and teachers of the patients, the researchers only invited the evaluators to assess and gave unified instructions but did not conduct face-to-face training. The reviewer consistency assessment cannot be completed, so the final score may be affected by the reviewer’s factors. In addition, whether the gender of the teacher affected the assessment results was not explored. The assessments of parents and class teachers who are not familiar with the patient at all are inaccurate, so we exclude their assessment data. However, it is a limitation of our research that we were unable to verify the effect of the three familiarity levels of completely unfamiliar, somewhat familiar, and very familiar on the evaluation results. Finally, although there are restrictions on the interval between parents and class teachers completing the assessment. However, patients commonly do not stay with their head teacher and parents at the same time, which can lead to observer bias in evaluating patients. These are limitations of this study.

## Conclusion

Limited to the relationship between the patient and the rater, the patient’s performance at the behavioral level or the perception of the parents/teacher may affect the scale score, suggesting that clinicians should consider information from multiple scenarios when using the scale to assess children’s behavior. At the same time, there are differences in the evaluation results based on different evaluation methods, which also suggests that patients should be evaluated by different assessment methods. In addition, patients with diagnosed ADHD were evaluated in this study, while fathers scored lower on several dimensions than teachers and mothers, so fathers do not seem to be an optimal evaluator. In general, when using the SNAP-V for assessment, it should be comprehensively considered from both the scorer and symptom dimensions. The study’s conclusions come from Chinese samples, and their generalizability across cultures needs to be confirmed by more studies.

## Data availability statement

The original contributions presented in the study are included in the article/supplementary material, further inquiries can be directed to the corresponding authors.

## Author contributions

XH and H-QL: conceptualization, methodology, writing-reviewing and editing, and writing original draft. AS: writing-reviewing and editing. J-JX: data collection, data analyses, and writing-reviewing. W-JT: methodology, data analyses, and writing-reviewing. Y-YL: conceptualization, methodology, writing-reviewing, and supervision. All authors contributed to the article and approved the submitted version.

## Conflict of interest

The authors declare that the research was conducted in the absence of any commercial or financial relationships that could be construed as a potential conflict of interest.

## Publisher’s note

All claims expressed in this article are solely those of the authors and do not necessarily represent those of their affiliated organizations, or those of the publisher, the editors and the reviewers. Any product that may be evaluated in this article, or claim that may be made by its manufacturer, is not guaranteed or endorsed by the publisher.
